# Generational differences in spatial mobility: A study with mobile phone data

**DOI:** 10.1002/psp.2210

**Published:** 2018-11-19

**Authors:** Anu Masso, Siiri Silm, Rein Ahas

**Affiliations:** ^1^ ETH Zürich, Switzerland; University of Tartu, Estonia; Talinn University of Technology Estonia; ^2^ Department of Geography University of Tartu Tartu Estonia

**Keywords:** activity space, generational differences, mobile phone big data, social acceleration, social transformations, spatial mobility

## Abstract

This article focuses on generational differences in spatial mobility. Assuming that the ability to cope with the social transformations related to growing mobility varies significantly across generations, we use mobile positioning data collected in Estonia during 2014 providing four main indicators, namely, the number of locations visited and the distances between visited locations, within Estonia and abroad. The results indicate that spatial mobility declines linearly with age; however, a high degree of heterogeneity exists within age groups. Whereas the spatial mobility of the most active members of the younger generation takes place mostly within Estonia, among the most active older generation focus their activity beyond its borders. The study reveals “delayed mobility” patterns among the most active groups of the older generation and a new “immobility culture” among the younger generation in terms of cross‐border activities in a transition society.

## 1 INTRODUCTION

One of the key processes in contemporary societies is the increase in spatial mobility, involving growth in the mobility of individuals, goods, knowledge, and information. Such changes have been generalised as the “mobility turn” in the literature (e.g., Cresswell, 2011; Faist, 2013; Urry, 2000) and referred to in discussions across multiple disciplines, including geography, social sciences, and migration studies. Studies have indicated that this increase in spatial mobility is directly related to a growth in the numbers of everyday activities for individuals and an acceleration of the pace of life, which has been referred to as social acceleration (Castles, 2010; Kaufmann, Bergman, & Joye, 2004; Rosa & Scheuerman, 2010). Many large‐scale changes in the behaviour of both societies and individuals are directly or indirectly related to rapid developments in information and communication technologies (ICT), which therefore shows the significant influence of ICT on spatial mobility (Green, 2002; May & Thrift, 2003). The current theoretical literature emphasises increased spatial mobility in relation to the spread of ICT in contemporary societies as one of the most essential social transformations of the present day (Kaufmann et al., 2004; Rosa 2010). However, not all individuals are able to keep up with these changes, which therefore results in problems such as a growing fragmentation of activities and pressure for “time‐appropriate behaviour” (Rosa, 2013).

In the present article, we contribute to the discussion of changes in spatial mobilities by studying empirically the generational differences in spatial mobility, as a means of studying social change. Only a few studies exist regarding age differences in spatial mobility and the geography of activities (Frändberg & Vilhelmson, 2011; Grotz, 2003; Langevang & Gough, 2009; Smith, Shaw, & Huckle, 1979); these approaches mainly concentrate only on the social‐chronological margins (the very young or the very old) and rarely consider connections and differences between generations. Although the relational geographies of age are hinted at in the theoretical literature (Hopkins & Pain, 2007), little empirical research has focused on these issues. Some authors have analysed changing (im)mobility practices in the context of social transformations (Meier & Frank, 2016), but there have been few attempts to analyse the ability of individuals to keep up with spatial transformations and the growing mobility among various generational groups.

The objective of the current study is to examine generational differences in the spatial mobility of individuals. We operationalise intergenerational differences in spatial mobility through the empirical study of spatial mobility in six age groups. We assume that members of age groups who were born and grew up in different sociocultural contexts with varying degrees of accessibility to spaces within and beyond their national territory will express different patterns of spatial mobility. Our empirical study was conducted in the rapidly transforming society of Estonia, where a variety of spatial transformations, including freedom of movement of people and an increased access to ICT and other mobility technologies, took place within a relatively short period at the beginning of the 1990s. Estonia is therefore an excellent example revealing the ongoing shifts in spatial mobility and contributing to their study. We assume that within particular age groups, the ability to cope with social transformations related to growing mobility may vary significantly. The empirical results of the differences within and between age groups, reflecting their cohort or life cycle effects regarding spatial mobility, are empirically examined and discussed using the intertwined theoretical approaches of individual lifetime, life course, and sociohistorical theory of generations. This offers a robust framework for explaining both changes in spatial mobility across generations in particular and sociospatial transformations in general. We aim to respond to the following research questions: (a) What kind of age differences can be observed in spatial mobility within Estonia and abroad? (b) What are the differences in spatial mobility between the most and least spatially active individuals?

## 2 THEORETICAL BACKGROUND

### 2.1 The spatial mobility of individuals

The spatial mobility of individuals denotes the use of space as a whole, including places visited and movements between these places. Spatial mobility is also closely related to time: All movements in space can be characterised by speed, and all visited places can be characterised by time spent in these locations. The spatial mobility of individuals can be summarised by their activity space, which incorporates all visited places during a certain time period (Golledge & Stimson, 1997; Schönfelder & Axhausen, 2003). Individuals' mobility can be restricted to a relatively compact area (measured by the area or ellipse of activity space) or can be expressed as a diffuse assemblage of visited places (measured by the number of places, the distance between these locations, and movement trajectories). Studies in this area usually focus on analysing “daily mobility” between activity locations^1^ as an essential part of everyday life. Some locations may be visited more regularly,^2^ such as the home and the workplace, whereas others, such as shops, sports facilities, the homes of friends, may be visited less often but still relatively frequently (Flamm & Kaufmann, 2006). Less regularly or randomly visited places might more commonly be connected with leisure activities. However, nowadays, the distinction between regularly and more randomly visited places is becoming vaguer, just as places are becoming more closely intertwined (as a result, for example, of the growing importance of working at home or on the move and completing household management‐related activities at work (Axhausen, Larsen, & Urry, 2006; Kwan, 2007; Urry, 2003).

Researchers generally agree that individuals now have higher spatial mobility than they did centuries or even decades ago, mostly due to better access to transportation technologies and changes in everyday practices. Instead of the classical modern form of mobility, where life was mostly centred around a small number of fixed activity places, late modernity is characterised by constant movement in everyday lives and an increase in the episodes of action and/or experience per unit of time. Although many of these changes occurred in Western countries, including a rise in cross‐border mobility and rapid increases in the numbers of personal vehicles, in postsocialist countries, the changes took place over a relatively short time period and somewhat later than in Western countries. Authors have therefore labelled the changes in spatial mobility in transition societies as a “delayed mobility turn” (Popov, 2012). Empirical studies have shown that cross‐border mobility can be a significant resource, especially for inhabitants of former Eastern bloc countries (Jamieson & Jamieson, 2002; Machacek & Lasticova, 2003). Therefore, spatial mobility can be one way to ensure access to higher levels of social status and income (Verwiebe, 2004) or to create a feeling of being “returned to the West,” as stated previously (Lauristin, 1997).

However, different groups cope differently with the increase in mobility, depending on the available resources. Studies have focused on three types of factors that explain variations in spatial mobility: external, socio‐economic, and human internal. External factors emphasised in the literature are numerous and include the existence of a personal vehicle (Kamruzzaman & Hine, 2012; Schönfelder & Axhausen, 2010), the number of working hours, the place of residence (Miranda‐Moreno, Eluru, Lee‐Gosselin, & Kreider, 2012), the social network (Axhausen, 2007; Carrasco & Miller, 2006; Lee & Kwan, 2011), and the use of ICT. The use of ICT has also been one of the central generative mechanisms explaining the increased frequency of spatial mobility, or the opposite, contributing to lower mobility due to the rise in mediated interactions (Thompson, 1999). Several studies have found that variations in spatial mobility can be explained by socio‐economic factors where, in addition to generational differences, gender, income, level of education, and occupation may also play a role (Kamruzzaman & Hine, 2012; Miranda‐Moreno et al., 2012), whereas others have shown that the effect of socio‐economic factors is rather small (Schönfelder & Axhausen, 2010). In the case of human internal factors, spatial mobility may depend on factors related to individual characteristics: habits, values, preferences, attitudes, prejudices etc. (Van Acker, Van Wee, & Witlox, 2010; Van Wee, 2009), which are often acquired in particular sociocultural contexts.

In summary, scholars agree that current societies are characterised by higher spatial mobility. However, it remains the case that not all individuals are able to keep up with the “pressure” to increase their spatial mobility.^3^ Analysing generational differences in spatial mobility offers an opportunity to explain the temporal changes and variations in spatial mobilities in particular and spatial changes as a form of social transformation in general.

### 2.2 Generational differences in spatial mobility

Previous studies have resulted in general agreement that age could be one of the essential factors explaining variations in spatial mobility. Nevertheless, the results of some empirical studies are contradictory, depending on the theoretical approaches used to explain the age differences. Differences in spatial mobility among age groups could be explained through three dominant theories: (a) the individual lifetime or life span approach, (b) the life course perspective, and (c) the sociohistorical theory of generations.

The individual lifetime approach sees age as a life span from birth to death, and chronological or developmental age as a stage in the general process of ageing. Empirical studies in this field focus mostly on external factors such as the availability of leisure activities (Mollenkopf, Hieber, & Wahl, 2011) and transport (Hjorthol, Levin, & Sirén, 2010) or housing amenities (Collins, Havens, & Tate, 2002) to enhance life quality. Studies concerning ageing as a process that may result in the loss of mobility due to health and functional abilities (Swiaczny, 2010) or social networks as possible facilitators of mobility (Litwin & Stoeckel, 2013) are less dominant in this strand of literature. However, such conceptions of generation are often criticised (Hörschelmann, 2011; Mannheim, 1997) because they promote a biological perspective and neglect social factors, the latter being more key to the other two approaches of life course perspective and sociohistorical theory.

The sociological life course approach focuses on human development and life experiences during different stages of life: childhood, youth, adulthood, middle age, and retirement. The social timetable of the life course is defined by the age criteria involved in social norms and roles (e.g., activity in labour market, parenthood, and retirement; Elder, 1975). This life course perspective is the one most commonly used in other empirical studies of spatial mobility. These studies indicate mobility decline over the life course (Frändberg, 2006) or different modes of mobility used across life cycles (Cass & Faulconbridge, 2015). Authors agree that children and the elderly tend to be based more in their home neighbourhoods, whereas people of working age are more mobile (Collins et al., 2002; Kraft, 2014). International mobility clearly declines from one's midtwenties (Frändberg, 2006) and is lower among the middle aged (especially among women; Pooley, Turnbull, & Adams, 2005). Spatial mobility (both international and domestic) may increase again in later life, foremost in relation to retirement (Berg, Levin, Abramsson, & Hagberg, 2014). It is therefore suggested that special attention should be given to transitional processes from one life stage to another, which involve periods such as retirement when new activity patterns are considered, evaluated, and practised (Berg et al., 2014). Certain life events such as marital status, household composition, or employment status may also influence an individual's spatial mobility (Geist & Mcmanus, 2008), and individual life courses are therefore also characterised by ruptures, returns, and delays (Hörschelmann, 2011).

The focal point of the third approach, the sociohistorical theory of generations, is that the birth year of individuals should be understood as their spatio‐temporal starting point for the explanation of their experiences in the context of general social change (Mannheim, 1997). According to this approach, age differences are attributed not only to an individual's “actual and potential participation in the social currents of their time and space” (Pilcher, 1994) but also to their “generational discourse concerning the general historical background of this birth cohort” (Corsten, 1999). Generational theory underscores intergenerational and intragenerational differences, distinguishing social generations and active generational units who may to varying degrees participate in and react to the events of the time (Mannheim, 1997). Although theories of spatial mobility have also suggested using generational perspective as an essential starting point (Kaufmann et al., 2004),^4^ few empirical studies in this field take into consideration the sociohistorical context of individual spatial mobility. Such studies as there are have indicated (Frändberg, 2006) that mobility experiences are acquired in the earlier stages of life through socialisation in a particular spatio‐temporal context and can be carried over to the following phases of life.

A number of authors have shown that differences in spatial mobility could be explained by a combination of these three approaches. For example, the interrelatedness of the personal life span and its location in sociohistorical time (Corsten, 1999; Hörschelmann, 2011; Mannheim, 1997) is emphasised in some theoretical approaches. Because the basic contours of our life world do not remain stable during an individual's life span, “the pace of socia change has increased from an inter‐generational pace in pre‐modernity to a generational pace in ‘classical’ modernity and further on to an intra‐generational pace in late modernity” (Rosa, 2005). For example, previous authors have indicated (Pooley et al., 2005; Westin & Vilhelmson, 2011) that the elderly of today generally enjoy lifestyles that are spatially more active than those of the elderly a few decades ago. Younger generations born between the 1980s and the early 2000s (Ozanne, 2012) may currently be less spatially mobile, their mobility habits being shaped by the wide technological developments around which they have grown up (Prensky, 2001). However, whereas in some countries, such as the United States, Canada, the United Kingdom, Norway, South Korea, and Japan, there are reports of a new immobility culture among younger generations, other countries such as Finland, Switzerland, Spain, and the Netherlands (Sivak & Schoettle, 2012) have seen no such change.

On the basis of these theoretical approaches and studies, we assume that a combination of personal life course approach and individual location in sociohistorical time should be taken into consideration to explain age differences in spatial mobility.

### 2.3 Sociospatial transformations

The sociohistorical theory of generations suggests that variations in spatial mobilities may be explained by taking into consideration individuals' experiences in the context of general social change.

Among the most significant factors explaining changes in spatial mobilities in transition societies are geo‐political changes, for example, the shift from a closed totalitarian to an open independent society around 30 years ago. During the Soviet period, spatial mobility was rigorously restricted in the Soviet Union, especially in areas close to the Western border. The Soviet period as an alternative form of cultural globalisation is also referred to in the literature as “failed spatial transition” (Masso, 2008a), because during this period, it was only ideological superstructures that were changed (e.g., “proletarian internationalism”), without clear manifestations in space (e.g., restricted opportunities for mobility and limited personal contact with Western countries). The period of rapid spatial transformation that began with the dissolution of the Soviet Union and Estonian independence in 1991 resulted in an opening of state borders and brought about accompanying cross‐border and intracountry mobility opportunities for individuals. The following posttransition spatial period can be characterised as a relative “calming down” of social space (Masso, 2008a), with the appropriation of the changed conditions of spatial activity taking place at the level of the individual.

The second essential factor explaining changes in spatial mobilities in postcommunist transition societies is the access to and spread of ICT technologies. In Estonia, the opening up in spatial terms took place in parallel with the rapid spread of ICT. The development of ICT is considered one of the most important technological revolutions in history and has changed human society immensely, with several researchers suggesting that society has not yet reached the highest level of technological development in ICT (Couclelis, 2009). At the same time, the effect of ICT on human behaviour and the functioning of society has been studied relatively infrequently, because the process is extremely rapid and complicated. Research questions and theories posed today often become outdated because the technologies and their use change radically by the time their study is complete (Aguiléra, Guillot, & Rallet, 2012; Kenyon & Lyons, 2007). Therefore, in order to study the expected and unexpected influences of ICT, new types of data are increasingly being used, where these allow for a more rapid data collection cycle and often provide a better overview of the influences of ICT.

Studies show that the development of ICT affects human activities in diverse ways, including reducing physical/geographical distance, speeding up information exchange, increasing spatial mobility, and social networks and networking (Larsen, Urry, & Axhausen, 2008). For example, during the Soviet era, individuals used various strategies, such as following Western media channels, to compensate for their spatial isolation. However, digital communication space is radically different from physical space; distances, temporal limits, and national boundaries are of less significance. New digitally mediated forms of residency and internationalisation are emerging, as exemplified by the Estonian e‐residency project (Tammpuu & Masso, 2018). The “temporal–spatial compression” caused by ICT has considerably increased the spatial mobility of people and accelerated how they use their time. For example, a single day now includes more activities, and there is more movement between these activities. Studies indicate that in transition countries, population groups with various “individual speeds” can be distinguished: those actively following and choosing their communication channels and those with rather one‐sided traditional habits regarding media and communication technologies (Lauristin et al., 2017). In addition, many everyday activities are developed by continuous information exchange without preplanning. All of this greatly affects issues of migration, travel behaviour, and environmental use and the rate of and geography involved in the mixing or separation of population groups.

ICT has intensified communication and information exchange between people and has significantly changed the geography and structure of social networks (Carrasco & Miller, 2006). Many researchers have noted that in social media, it is easier to overcome various social barriers (e.g., those based on gender, ethnicity, and education) and to find people with similar interests and views around the world. This in turn results in changes in social ties, labour markets, entrepreneurship, cultural spaces, migration, crime, and many other aspects of everyday life. However, not all individuals have equal access to ICT; the digital divide between generations is one of the clearest consequences of rapid technological change. Although the technology‐friendly nature of Estonian society has been highlighted (e.g., Tammpuu & Masso, 2018), other studies have indicated that individuals in transition societies have adjusted to these spatial and ICT transformation processes to varying degrees, such that a personally “globalised” group can be distinguished from another that feels uncertainty in relation to spatial opening, digital media technologies, and globalisation (Kalmus, Masso, & Lauristin, 2013; Masso, 2008b).

## 3 DATA AND METHODS

### 3.1 Passive mobile positioning data

To assess the spatial mobility of various age groups, we use passive mobile positioning data, which provide the locations of the call activities of individuals during the year 2014. Previous studies (Järv, Ahas, & Witlox, 2014; Silm & Ahas, 2014) have shown that the locations of call activities recorded during the course of a longer observation period (1 year) can be used relatively reliably to describe key anchor points and the spatial mobility of individuals.

Passive mobile positioning data are automatically stored in the memory or log files held by mobile phone operators. Data used for this study consist of call detail records (CDR) from the billing database of the largest Estonian mobile phone operator, Telia (Ahas, Aasa, Roose, Mark, & Silm, 2008). Telia is a market leader in Estonia with an estimated 39% of market share in 2015 (Tehnilise Järelvalve Amet, 2015), and approximately 94% of the Estonian population have access to mobile phones (Eurobarometer, 2013). Telia's network covers 99.9% of the country.

We analyse two types of passive mobile positioning data in this study; the first database covers call activities of the residents of Estonia while in Estonia. The database consists of the locations of outgoing call activities (outgoing calls and text messages sent) via mobile phones via the Telia network. For each call activity, the database includes the randomly generated ID number of the phone used, the time at which the call activity was made (to an accuracy of 1 s), and the location (to the accuracy of the network cell). The second database covers the call activities of residents of Estonia while abroad (i.e., travelling outside of Estonia). The database contains the incoming and outgoing calls and text messages sent abroad via Telia's roaming service and contains the ID number of the phone used, the time at which the call activity was made (to an accuracy of 1 s), and the location (to the accuracy of the country) for each call activity. The ID numbers of the phone users are identical for each phone user for the whole period of the database (provided that the contract of the phone user did not change) and also across the two databases, which enables us to connect them. The assigned ID ensures anonymity and cannot be associated with a specific individual or phone number. In addition to call activities, the gender, birth year, and user language of the mobile phone users were provided by Telia, for research purposes only. These details are not available for all phone users: At least one of the characteristics is available for 37% of the phone users and all three for 22% of the phone users. The user language (Estonian, Russian, and English) is the language of communication with the operator. In addition, the CDR data are used to identify the home‐ and work‐time anchor points for each user, using a model based on the location and the timing of the call activities of each user over a 1‐month period (Ahas, Silm, Järv, Saluveer, & Tiru, 2010).

The collection, storage, and processing of the data obtained from Telia comply with European Union requirements regarding the protection of personal data as per EU directives on handling personal data and the protection of privacy in the electronic communications sector through the general data protection regulations (European Parliament and Council of the European Union, 2016). Approval for the use of the data was also obtained from the Estonian Data Protection Inspectorate.

### 3.2 Methods

The study covers the 1‐year period from January to December 2014 and involves people who met the following criteria: aged 20 years and older, language Estonian or Russian, known gender, place of residence, and call activities for the entire study period (each 12 months). Individuals younger than 20 and those using English as their main language of communication with the mobile phone company were excluded from the data because their absolute share was marginal^5^ (*n* < 50) and was therefore too small to allow comparisons with other age groups in the multivariate statistical analysis. Our study therefore focused only on adults who made their own decisions about particular activities and mobility in space. A total of 95,489 people met these criteria and were included in the present analysis.

In order to analyse intergenerational differences in spatial mobility, the sample was split into six groups representing age cohorts, rather than forming clear‐cut social generations as per Mannheim's (1997) interpretation. These cut‐off points were chosen for statistical reasons, constituting numerically comparable groups suitable for multivariate statistical analysis and ensuring confidentiality by merging moderately represented groups (e.g., 70 and over). We also considered that the use of generation as a socially constructed concept depends, according to various theoretical perspectives (e.g., Mannheim, 1997), on the existence of a shared generational identity. Other empirical studies on the perceived length of a generation in years have shown that the largest group of respondents perceived 10 years to be the most appropriate size for their generation (Kalmus, Masso, & Lauristin, 2017). Based on these considerations, we constructed the age groups as follows:

- 20 to 29 year olds (born between 1985 and 1994; mostly born and growing up in the period after Estonia regained independence; mainly students or those in the initial phases of their careers);
- 30 to 39 year olds (born between 1975 and 1984, with their formative years falling in the period towards the end of the Soviet era or after Estonia regained independence; mainly engaged in working and raising children);
- 40 to 49 year olds (born between 1965 and 1974, who experienced diverse social circumstances during their formative years; mainly in the active phases of their careers);
- 50 to 59 year olds (born between 1955 and 1964; their formative years coincided with the Soviet period; mainly engaged in work);
- 60 to 69 year olds (born between 1945 and 1954; their formative years coincided with the Soviet period; mainly engaged in work but many of them pensioners);
- 70+ year olds (born 1944 or earlier; their formative years in the Soviet period, mostly economically inactive pensioners).

As shown in Table 1, the sociodemographic profile of the database differs slightly from that of the population of Estonia as a whole, based on 2011 census data (Statistics Estonia, 2011), such that the youngest (20–29) and oldest (70+) age groups are somewhat under‐represented, and the middle age groups (40–49 and 50–59) are over‐represented in our study.^6^ The proportion of women is a little higher, and the proportion of men is accordingly somewhat lower in the mobile phone database compared with the population. The biggest difference can be seen in the case of language spoken, so that Estonian speakers were over‐represented and Russian speakers under‐represented in the mobile phone database. However, we note that the mother tongue reported in the national census does not necessarily reflect the actual use of language in everyday situations such as communicating with the mobile phone company, which could explain the differences in the table, along with differences between language groups in preferred mobile phone companies.

**Table 1:** Distribution of characteristics of the individuals in the study compared with the population as a whole (%)

	Mobile positioning	Census 2011^a^
Age		
20–29	3	18
30–39	18	17
40–49	31	17
50–59	24	17
60–69	14	14
70+	10	17
Gender		
Male	41	46
Female	59	54
Language^a^		
Estonian	86	68
Russian	14	32
Total	100	100

a Language chosen when signing a contract with the mobile phone company in the case of mobile positioning data, and mother tongue in the case of national census.

In order to estimate spatial mobility among the six chosen age groups, we analyse two dimensions: number of locations visited and the distance between visited locations, based on the theoretical considerations we have previously explained suggesting the importance of both distance and frequency. These two dimensions are measured both within and outside Estonia, allowing us to use four indicators characterising a person's spatial mobility (Table 2). The indicators are calculated on the basis of single measured variables to enable better generalisations across age groups. Indicators that show the number of visited locations enable us to estimate the intensity of individuals' spatial mobility by reference to the number of unique activity districts visited, and the number of anchor‐point districts within 3 months and for more than 3 months within Estonia, or the number of trips and number of countries visited abroad. Indicators that show the distances between visited locations enable us to study the spread of spatial mobility, based on the average distance of unique activity districts from the place of residence and monthly average distances between work and place of residence within Estonia, or the distance between capital cities of the countries visited abroad. The distance between capital cities was chosen because the data make it possible to locate foreign trips only to the accuracy of the country concerned, and the location of the capital seems a better indicator of location than the centre of the country, for example, and was also previously shown to be an effective metric (Nilbe, Ahas, & Silm, 2014). The unique activity districts are those in which a person made at least one call activity during the study period. Anchor‐point districts are those where call activities were made on at least two different days in any particular month (Ahas et al., 2010) and in a minimum of 3 months per year. We used the district level because the accuracy of the CDR data is 100–1000 m in cities, in more densely populated areas and in areas with denser networks of roads, and 1.5−20 km in more sparsely populated areas (Ahas et al., 2008). We therefore had to use areas greater than the coverage areas of mobile antennas. The location is based on the location of the mobile antenna where the call activity was made. The districts are local municipalities and separate municipal towns, boroughs, and city districts in Tallinn, comprising 240 districts altogether. The indicators characterising locations outside Estonia (Indicators 3 and 4 in Table 2) involve only those individuals who left Estonia for a time and therefore completed all their call activities abroad.

**Table 2:** Indicators and underlying variables used for analysing spatial mobility

Indicators	Variables
1. Number of locations visited within Estonia	1.1. Number of unique activity districts
	1.2. Number of anchor‐point districts for 3 months and over
2. Distance between locations visited within Estonia	2.1. Average distance of unique activity districts from place of residence
	2.2. Monthly average distance between work and place of residence
3. Number of locations visited outside of Estonia	3.1. Number of trips abroad
	3.2. Number of countries visited
4. Distance between locations visited outside of Estonia	4. Distance between capital cities of the countries visited abroad

The values of each of these single variables (1.1. to 4 in Table 2) were divided into quartiles where the lower quartile indicates less activity regarding a particular dimension of spatial mobility. Next, the values of these variables were summed up to produce four indicators (1–4 in Table 2). The aim of this recoding was to standardise the variables prior to the generation of the index, increasing their comparability in situations where the number of underlying variables differs somewhat across indicators (e.g., in the case of Indicator 4, only one underlying variable is used, whereas two underlying variables are used in other indicators).

The analysis indicates a high level of internal consistency of the indicators, in that the underlying variables of a particular indicator are strongly related (Cronbach's alpha >0.7). The results of classification techniques, including a factor analysis, also suggest merging the initial variables into four factors. There are also significant correlations between indicators that measure spatial mobility within (*r* = 0.496) and outside Estonia (*r* = 0.261), indicating the importance of the geography of the spatial mobility and suggesting the division of indicators into domestic and international spatial mobility.

To analyse differences in spatial mobility among different age groups, the values of the indicators were assessed using a general linear model

$$Y_{i}=a+\sum_{k=1}^{k}\;\beta_{k}\chi_{\mathrm{ik}}+C_{i},$$

where *Y*_*i*_ is the value of the indicator by individuals *i* = 1, …, *n*; α is a constant; *χ*_*ik*_ is the value of the variable for an individual; *β*_*k*_ is the parameter describing the effect of this variable, with *k* variables; and ϵ is the error term. We used the full model, which includes all variables in addition to age; thus, it also included gender, language, number of residential districts, settlement system hierarchy level, and number of call activities per day. Similar models were constructed for all four indicators (Models 1 to 4 in Table 3).

**Table 3:** Spatial mobility by age, controlled by background variables

	General linear Model B, significance				Binary logistic regression model Exp (B), significance
	Model 1. Number of locations visited within Estonia^a^	Model 2. Distance between locations visited within Estonia	Model 3. Number of locations visited outside Estonia	Model 4. Distance between locations visited outside Estonia	Model 5. Visits outside Estonia^a^
Age 20–29 (ref. 40–49)^b^	0.225***	0.154***	0.009	−0.005	1,238***
30–39	0.049***	0.021**	−0.048***	−0.065***	1,017
50–59	−0.022***	−0.039***	−0.018	0.076***	937***
60–69	−0.286***	−0.233***	−0.159***	0.142***	613***
70+	−0.532***	−0.400***	−0.322***	0.102***	266***
Gender female (ref. male)	−0.196***	−0.026***	−0.122***	0.186***	991
Language Russian (ref. Estonian)	−0.455***	−0.337***	0.008	0.314***	1,023
Number of residential District 2 (ref. 1),	0.389***	0.616***	−0.004	−0.013	1,098***
3 and more	0.921***	1.078***	−0.027*	−0.040***	1,090***
Settlement system hierarchy levels: Regional centres and commuting area (ref. capital city and commuting area)	−0.393***	0.328***	−0.216***	−0.227***	572***
Small towns and rural areas	−0.201***	0.156***	−0.310***	−0.472***	412***
Number of call activities per day: 0.2–1.9 (ref. 2.0–2.9)	−0.638***	−0.285***	−0.263***	0.014	566***
3.0–6.9	0.550***	0.179***	0.157***	−0.004	1,425***
7.0–41.8	1.095***	0.359***	0.341***	−0.021	2,059***
Intercept	2.492	1.811	2.666	2.460	58,788
Goodness‐of‐fit^c^	0.472	0.225	0.055	0.047	163

a The reference category is “does not make any visits outside of Estonia.”

b Middle age group is used as the reference category, because it is generally homogeneous unlike the younger and older age groups in the comparison, thus facilitating the interpretation of results.

c *R* ^2^ was used in Models 1–4, Nagelkerke in Model 5.

* Significant at 0.1.

** Significant at 0.05.

*** Significant at 0.01.

In addition, the probability of visits outside of Estonia was assessed using the binary logistic regression model

$$\text{log}\;\frac{\rho \left(V_{i}=1\right)}{\rho \left(V_{i}=0\right)}=a+\sum_{k=1}^{k}\beta_{k}\chi_{\mathrm{ik}},$$

where *ρ*(*Y*_*i*_ = 0) is the probability that individuals *i* = 1, …, *n* do not make visits outside of Estonia and *ρ*(*Y*_*i*_ = 1) is the probability that individuals *i* = 1, …, *n* make visits outside Estonia (Model 5). Using each indicator, we differentiated the most and least active individuals. The most spatially active individuals are those who have a value of 4 for a certain indicator (the highest quartile) and the least active have an indicator value of 1 (the lowest quartile). Spatially, the most and least active individuals are compared with those having average spatial mobility (second and third quartiles combined) through a binary logistic regression model

$$\text{log}\;\frac{\rho \left(Y_{i}=1\right)}{\rho \left(Y_{i}=0\right)}=\alpha +\sum_{k=1}^{k}\beta_{k}\chi_{\mathrm{ik}},$$

where *ρ*(*Y*_*i*_ = 0) is the probability of individuals *i* = 1, …, *n* belonging to the average spatial mobility (second and third quartiles) group and *ρ*(*Y*_*i*_ = 1) being the probability of individuals *i* = 1, …, *n* belonging to the spatially most active (fourth quartile) or least active (first quartile) group. We present the full model where in addition to the main independent variable of age, all available control variables are included. Similar models were constructed using all four indicators as dependent variables. In the models, the most and least spatially active groups were compared with those having average spatial activity. Such comparisons enable us to distinguish between those able to keep up with the prevailing mobility norm in society (the most mobile) and those unable to do so and lagging behind with regard to the average spatial mobility norm (the least mobile).

We also analysed the geographical differences in districts visited in Estonia by various age groups. This analysis was undertaken only for those individuals living in the capital city of Tallinn in order to avoid the predominance of place of residence on the maps presented.

## 4 RESULTS

### 4.1 Spatial mobility by age

**Figure 1:**
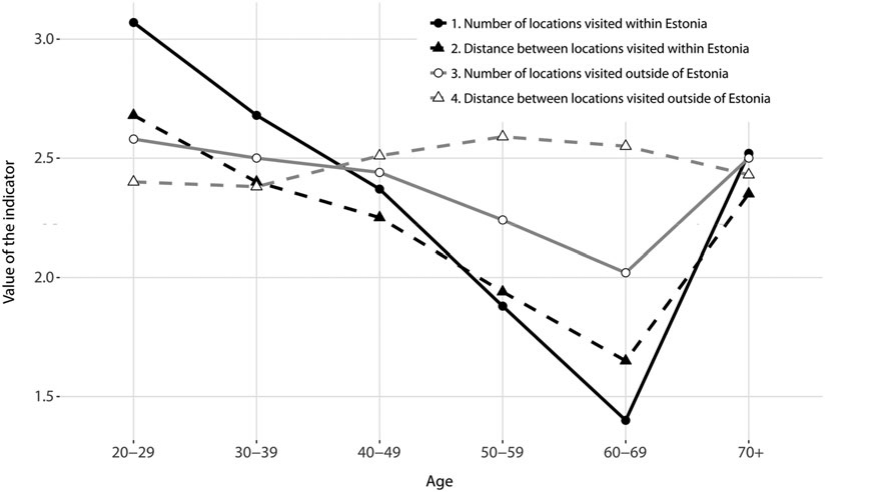
Spatial mobility by age (averages of calculated indicators on a scale from 1 [*lowest*] to 4 [*highest*] index value)

The results of the analysis show that there are significant differences in the spatial mobility of the six age groups analysed. Generally, spatial mobility declines linearly with age, being the highest among 20‐ to 29‐year‐olds and declining until the age of 60–69 (Figure 1). After a pronounced decline at the age of 60–69, spatial mobility increases sharply among the oldest age group (70+).

However, this tendency of shrinking spatial mobility until age 60–69, followed by sudden growth among the oldest age group, is seen mainly in the case of activities within the national territory, whereas spatial mobility abroad is more stable among different age groups. It should be borne in mind, however, that only those individuals who have travelled abroad are included in the analysis. The analysis thus shows spatial mobility outside Estonia rather than the frequency of trips abroad.

The younger middle age (30–49) and oldest age groups (70+) have quite similar spatial mobilities and are almost equally active both within and outside of Estonia. For the youngest age group, most of their activity is expressed in Estonia‐related mobility. The relatively large gap between the two indicators—the number of and distance between visited locations in Estonia—implies a higher degree of activity within a smaller spatial territory. The older middle age groups (50–69) have a higher spatial mobility outside Estonia than the youngest age group.

Regression analysis (Table 3) confirms the results of the descriptive analysis indicating significant differences in spatial mobility among various age groups. The age differences are significant even after controlling for available background variables, such as gender, language, number of residential districts, settlement system hierarchy levels, and number of call activities. The regression analysis in the table indicates that the youngest generation has the highest level of mobility within the national territory, both in terms of the number of visited locations and the distance between them (see Models 1 and 2 in Table 3). In contrast, the two oldest generations show more mobility abroad, but only in relation to the distance between visited locations, whereas the number of visited locations remains moderate.

However, when analysing activities outside the national territory, we note that Models 1–4 include only those who were abroad during the 1‐year period of interest. We therefore formed an additional Model 5, which also includes those who did note leave Estonia during the period. On average, about half the individuals in our study completed call activities abroad and thus left Estonia during the study period (49%). Model 5 in Table 3 indicates that older age groups have a lower probability of going abroad. On closer inspection, it appears that visits abroad generally decrease linearly with a person's age, so that the proportion of individuals who went abroad during the study period is higher among younger and middle age groups (64% among 20–29, 58% among 30–39, 55% among 40–49, and 51% among 50–59 age group) and lower among older age groups (37% among 60–69 and 18% among 70+).

In addition, the analysis presented in columns 1–4 indicates that men generally have higher spatial mobility than women. The only exception is the distance of the locations outside Estonia, meaning that among those who are internationally active, women tend to visit more distant countries than men. Individuals with multiple residencies have higher domestic mobility, as a result of commuting between these locations. However, these “multiresidents” do not show higher levels of mobility abroad, and the related domestic spatial mobility is not transferred to cross‐border mobility. Individuals living in the capital city have higher spatial mobility both within and outside Estonia. However, the distances involved in their activity within Estonia are shorter for residents of the capital than they are for those living outside the capital, probably due to the better infrastructure for both work‐ and leisure‐related activities in the capital.

### 4.2 Differences between the most and least spatially active individuals

The previous analysis indicates that in addition to differences in spatial mobility between age groups, there is also considerable variation within age groups. Therefore, for the following analysis, we distinguished between the most and least spatially active individuals.

**Figure 2:**
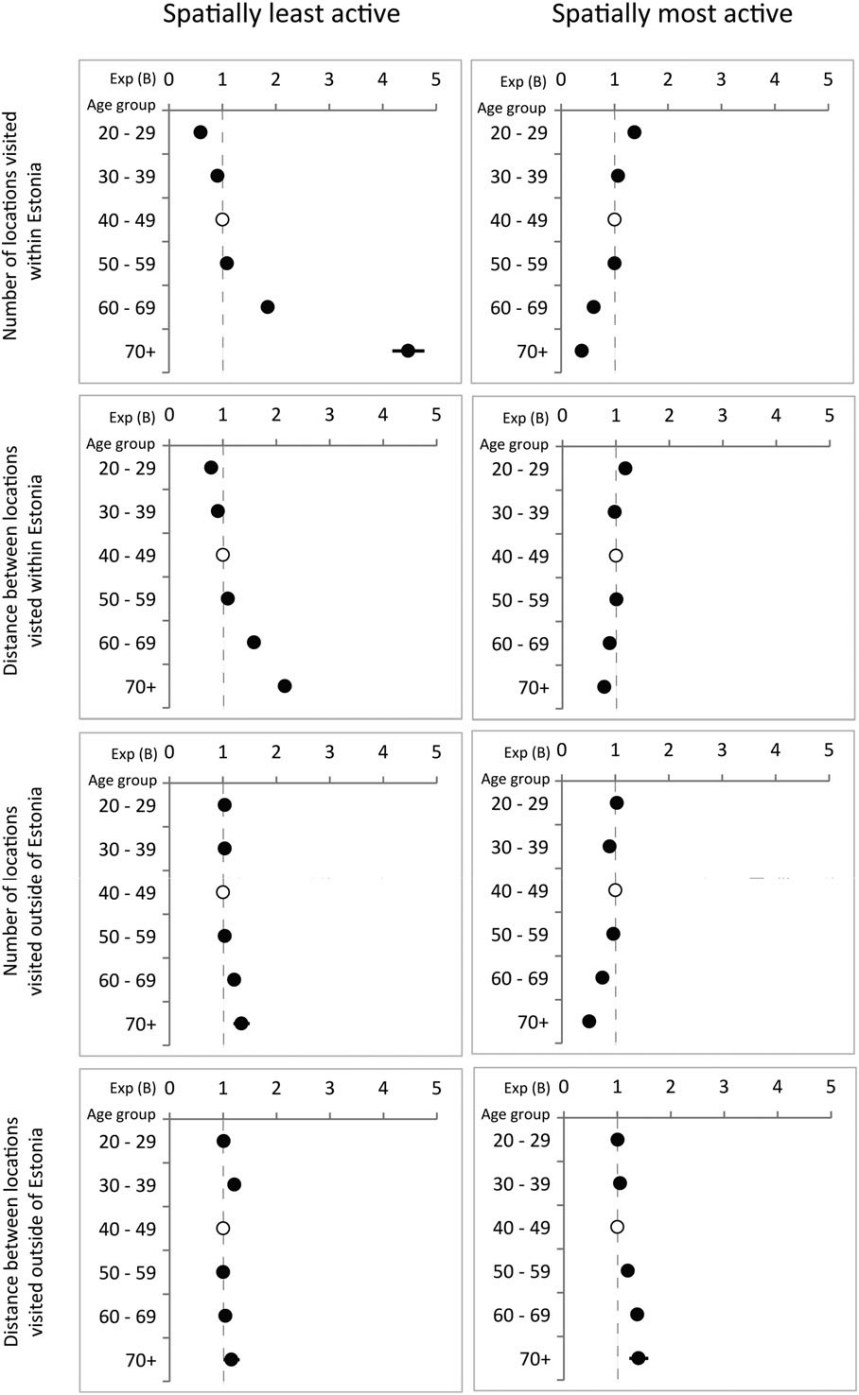
Comparison of most active and least spatially active individuals (binary logistic regression, exponential Beta)

The results in Figure 2 indicate that the most spatially active are the youngest (20–29) age group. Compared with the middle‐aged reference group, this youngest and most active group has a high level of mobility within Estonia, both in the number of and distance between visited locations. The next age group of 30–39 has a similar level of spatial mobility within Estonia, but only regarding the number of locations visited, whereas the distances between activity districts are lower. This 30–39 group is spatially less active in terms of the distances involved in visiting locations outside Estonia. The older middle age group (50–59) does not differ much from the reference group (40–49), both groups being spatially less mobile in Estonia but showing increased activity abroad in terms of the distance between visited locations.

The older age groups are distinguished by their high spatial mobility, but unlike the case for younger age groups, their spatial mobility is expressed mainly outside Estonia and in the distance between visited locations. In the spatially most active group, the distances of locations visited abroad increase with age, being the highest among the oldest age group. However, a relatively high level of heterogeneity characterises the cross‐border mobility of the oldest age group (70+). Although the spatially most active group with regard to the distance between locations visited outside of Estonia contains a higher proportion of the oldest age group than of other age groups, furthermore, this oldest age group is also statistically better represented in the spatially least active group, both in the number of and distances between visited locations and considering mobility both within and outside the national territory.

### 4.3 Geographical differences in activity locations

**Figure 3:**
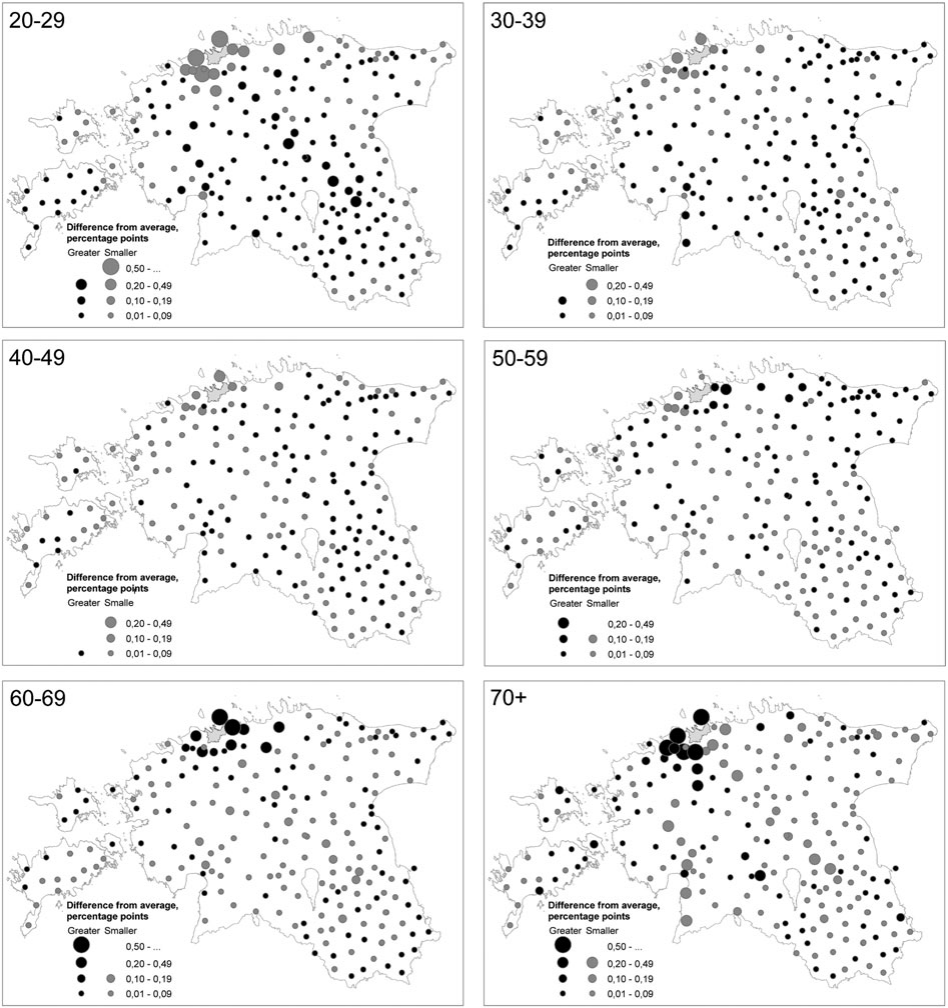
The relative distribution of districts visited in Estonia by various age groups. The averages of the indicators for each group were calculated, then the deviations from the averages (positive and negative) within each age group. Figures show the districts visited by individuals living in the capital city only

The spatial mobilities of various age groups are significantly distinguishable by the geography of districts visited, both within and outside Estonia. An analysis of the relative distribution of visited districts within Estonia indicates that the spatial mobility of younger age groups (mostly 20–29) is more widespread than for other age groups, such that they visited a greater number of districts during the study period (Figure 3). For middle age groups (30–49), the relative distribution of visited districts is similar to the average across age groups. People aged 50–59 more often visit districts in the east of Tallinn and in north‐eastern Estonia. The two oldest age groups have to some degree more limited spatial mobility (especially 70+), because their activities are more concentrated in or around their place of residence (in this case, Tallinn).

**Figure 4:**
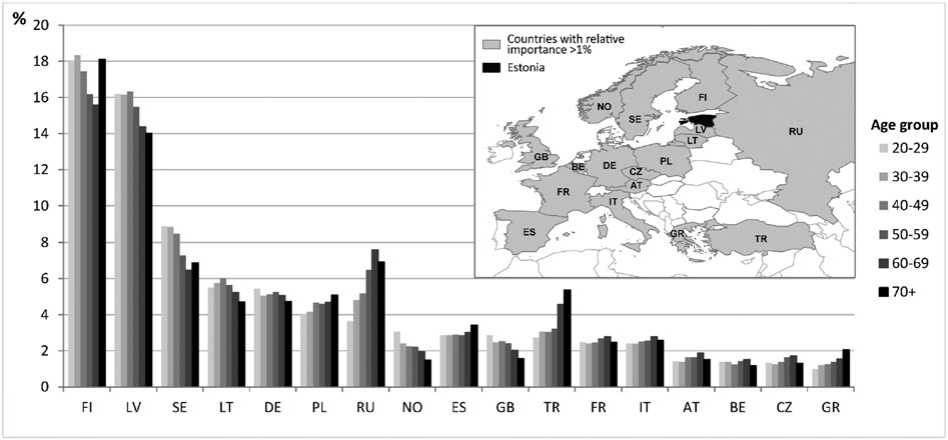
Relative importance of countries by age group. Only countries with relative importance >1% are presented in the figure. Every individual is represented in a given country only once: FI, Finland; RU, Russia; IT, Italy; LV, Latvia; NO, Norway; AT, Austria; SE, Sweden; ES, Spain; BE, Belgium; LT, Latvia; GB, Great Britain; CZ, Czech Republic; DE, Germany; TR, Turkey; GR, Greece; PL, Poland; FR, France

Mobility outside the national territory is also spatially divided across various age groups (Figure 4). Three age‐specific visited locations outside Estonia can be distinguished here. First, spatial mobility within Latvia and Great Britain (but also Norway) linearly decreases with age so that it is mainly the younger generations who visited these countries during the study period. A distinguishing feature of the youngest age group is also the high incidence of visits to the United States.

Second, visits to former postsoviet countries such as Russia, Poland, or the Southern‐European countries of Spain, Turkey, and Greece (and to some degree also France) increase linearly with age, such that older generations visit these countries most often. The increase in visits to these geographically more distant countries could also explain the mobility of older generations regarding the distance of visited locations abroad, as discussed previously. Another distinguishing feature of older generations is their visits to former soviet countries such as Hungary, Slovakia, and Bulgaria, where younger age groups go only rarely (<1%).

A third group of countries—Finland and Sweden—is similarly often visited both by younger and older age groups, whereas the middle age group appears to visit these places more seldom. The only country where the middle age groups are most active compared with other age groups is Lithuania. Another distinguishing feature of 30‐ to 39‐year‐olds is their attraction to Egypt, where other age groups hardly visit at all (<1%). Regarding other visited countries, the differences across age groups are statistically significant but very small(e.g., Germany and Belgium).

## 5 DISCUSSION AND CONCLUSIONS

This article has focused on spatial mobility as one of the most important constituents of everyday life in modern societies. Previous studies of age differences in spatial mobility have focused mainly on the sociochronological margins, without studying how spatial mobility varies across all generational groups (Frändberg & Vilhelmson, 2011; Grotz, 2003; Langevang & Gough, 2009; Smith et al., 1979). The aim of this article was to contribute to filling this gap by studying generational differences in spatial mobility, in order to explain changes in spatial mobility in modern societies.

The theoretical assumption (Castles, 2010; Rosa, 2005) that spatial mobility patterns in the context of rapid spatial transformations may vary generationally was confirmed in our analysis. Our previous assumption was that in a transition country, the socialisation of generational groups during periods with varying sociospatial conditions could explain differences in spatial mobility. However, the true picture is more complex, considering both the differences between and within age groups. As indicated in previous studies (Corsten, 1999; Hörschelmann, 2011; Mannheim, 1997), these generational differences and variations in spatial mobility can be explained by the interrelatedness of personal life span and location in sociohistorical time.

In comparing differences in spatial mobility between generational groups, our analysis shows decreasing mobility until the age of 60–69, with a sudden increase after retirement. The growth in mobility among the oldest age groups can be explained by the increase in leisure activities at a later age, the leisure activities being somewhat less spatially restricted than work‐related activities. On the basis of previous studies (Pooley et al., 2005; Westin & Vilhelmson, 2011), we also suppose that the elderly today enjoy somewhat spatially more active lifestyles than the elderly of some decades ago. A decline in spatial mobility with age until retirement was also found in previous studies (Frändberg, 2006) and can be explained by the life course effect. Younger age groups probably undertake more work‐ and leisure‐related activities, whereas the spatial mobility of older generations may be reduced due to decreases in work‐ and leisure‐related activities and narrowing personal networks.^7^ The low involvement of middle‐aged groups in international mobility also can be explained by their life courses and parallel involvement in work‐ and family‐related commitments. Such results thus show the existence of age inequalities (Woodman & Wyn, 2015) and age‐related residential segregation (Winkler & Klaas, 2012) among the least active group of older individuals whose everyday activities are somewhat more limited to their places of residence. However, unlike in previous studies (Kraft, 2014), we show that middle‐aged groups are not necessarily better integrated with higher mobility, because their international mobility is lower. Furthermore, unlike previous studies (Frändberg & Vilhelmson, 2011) pointing to a decline in cross‐border activities after age 20, our findings indicate that any decline in spatial mobility with age is seen only in the case of mobility within the national territory, with mobility outside the national territory being somewhat more similar among different age groups. One of the explanations for this might be that cross‐border mobility is mostly leisure‐related and therefore less dependent on life course events but is more dependent on individual lifestyle or on available economic, social, and cultural resources.

Besides differences between generations, there were also remarkable variations within age groups. In our analysis, we distinguish between the most and least active groups, making it possible to examine in more detail the patterns of spatial mobility for those “falling behind” or “moving ahead” of their contemporary patterns of mobility and in their ability to respond to the contemporary norm of mobility. These variations in spatial mobility, especially in the case of cross‐border mobility, can be explained by socio‐economic factors, especially by self‐estimated social status in the society, as indicated in previous studies (Masso & Opermann, 2017). In this study, we assume that such a distinction enables us to study in greater depth changes in spatial mobility as one of the main social transformations in postcommunist societies and thus take into account the possible tendencies and variations of socioculturally acquired mobility patterns.

Our findings show the degree of heterogeneity in spatial mobility, especially with regard to the focus of older generations on cross‐border activities. Although previous studies have indicated that younger and older generations may resemble each other regarding high spatial mobility (e.g., Kraft, 2014), this study enabled us to distinguish two different generational mobility patterns: The most active members of younger generations have a local focus in their activities, whereas the most active members of older generations have a more international focus in their activities. Such results in some way contradict our initial assumption based on the theory of social generations (Mannheim, 1997; Pilcher, 1994), in which younger generations born and raised in the period after Estonia regained independence had been socialised in the context of open state borders and therefore would show higher spatial mobility. This initial assumption was only partly confirmed, in that the spatially most active younger generations (born between 1985 and 1994) mainly expressed their activity in within‐country mobility. The more limited international mobility of younger generations might be explained by economic constraints or by alternative practices (e.g., the use of social media) that compensate for fewer direct international contacts. The smaller number of cross‐border contacts may also be explained by time factors: The younger generations have not had sufficient opportunities to broaden their networks beyond close family and friendship‐related ties.

For individuals from older age groups (60+) who expressed higher levels of spatial mobility, the distances involved in visiting locations abroad were significantly higher than among younger generations. Previous studies showed some increase in spatial mobility accompanied by new mobility practices among pensioners (Westin & Vilhelmson, 2011). In addition, the examination of the geography of places visited by various generational groups makes it possible to discuss some possible generational features of spatial mobility. For example, older generations (born between 1945 and 1954 or earlier) still preferred visiting some postcommunist countries, because they were culturally more familiar with those countries due to their access to them during the Soviet era, the period when they were growing up^7^. In addition, several individuals, especially from the Russian‐speaking population, may have had relatives and other types of contacts in the former Soviet Union and therefore visited those countries more often (Masso & Tammpuu, 2017). On the other hand, the higher interest in visiting western and southern European countries by these older generations compared with other age groups may reflect their wishes to “compensate” for their scarce experiences with these countries due to the spatial restriction during the Soviet era.

This study also aimed to examine and explain changes in spatial mobilities across generations, taking into consideration the hypotheses of previous studies regarding the increase in spatial mobility and growth in the number of everyday activities, leading to social acceleration in the acceleration of life pace (e.g., Rosa & Scheuerman, 2010). We revealed various individual abilities to cope with social acceleration and with the contemporary social norms of spatial mobility. We thus showed that certain generational patterns of “delayed mobility” among older generations (Popov, 2012) and a new “immobility culture” among younger generations (see also Berg et al., 2014) are on the increase in the transition society. The “digital divide” of generations, where younger age groups enjoy greater access and capital to use digital media technologies (Kalmus et al., 2013; Kalmus, Masso, Opermann, & Täht, 2018), giving them access to spatial locations, necessary interactions, and networks, could explain their relative spatial immobility. We assume that online activities decrease spatial mobility, through replacing face‐to‐face real‐time contacts with those mediated by communication technologies. However, the role of the media and communication technologies might also be one of the factors explaining the growing mobility among older generations, with information and communication technologies supporting the readiness for cross‐border mobility, in that they make it possible to obtain information about distant locations and therefore reduce any feeling of cultural insecurity (Masso, 2007; Masso & Opermann, 2017; Masso, Opermann, & Eljand, 2014). The assumption made in previous studies that older generations lead an increasingly “anachronistic life” (Rosa, 2005), their contemporary spatial mobility being appropriated or carried over from earlier periods, was found to be the case for only one group of spatially least active older (70+) people, whereas the other group showed high activity in cross‐border mobility. Therefore, the individual spatial capitalisation expressed in higher spatial mobility (Kaufmann et al., 2004) varied not only across but also within generations. The analysis reveals that developments in mobilities in transition countries where the “opening up” happened somewhat later and over a shorter period do not necessarily follow the same patterns as they do in Western countries, where the conceptualisations of “mobile generations” (Ozanne, 2012) or the “new mobility culture” (Hopkins & Stephenson, 2014) were formulated.

This study is not without limitations. First and foremost, it uses data obtained from the mobile positioning method, in which spatial behaviour is assumed to depend directly on mobile phone usage. Therefore, the accuracy of the spatial mobility measurements for all age groups may differ across the four calculated indicators within or outside the national territory, or regarding the number of and distance between visited locations. Similar to previous studies (Yuan, Raubal, & Liu, 2012), our findings indicate that movements recorded using mobile positioning methods were to some degree more random and less predictable among the youngest age groups (i.e., call activity and spatial mobility indicators were weakly correlated, *r* = 0.18, *p* < 0.001, compared with other groups on average, *r* = 0.21, *p* < 0.001) and regarding the distances involved in visiting international locations (*r* = 0.02 on average, the association being statistically nonsignificant compared with locations visited in Estonia, *r* = 0.45, *p* < 0.001). However, further study is needed to determine in more detail the associations between spatial mobility and calling activity and to explain the possible location‐dependent behavioural patterns of phone use (e.g., the elderly preferring to call from their homes).

Several additional issues may emerge when studying spatial mobility based on the mobile positioning method among those from the youngest age groups, who may be under‐represented in that they may use additional means of communication such as social media, apart from calling or sending messages via mobile phones. Additionally, the effect of digital inequalities might have been more obvious in older age groups, so that in our study, the spatially more passive individuals in the oldest age groups could have been excluded from the study because they do not use mobile phones. Further studies could therefore focus in more detail on a mixed‐method approach, combining mobile positioning data with survey data or social media sources in order to study generational patterns in spatial mobility. A more detailed longitudinal comparative analysis of individuals' mobility patterns would also make it possible to contribute to the field of population geography, by explaining whether the spatial mobility boundaries between age groups are changing, and if so, how. Further studies could also focus on a detailed comparative examination of the mobile positioning method with previously more traditional survey methods or with novel methods for measuring spatial mobility based on social media data. Nevertheless, the mobile positioning method described here offers a novel and valuable resource for studying actual patterns of spatial mobility and changes in mobility behaviour in different spatio‐temporal social contexts.

## CONFLICT OF INTEREST

The authors have no conflict of interest to declare.

## ENDNOTES

1 This has also been referred to as the “usual environment” in the field of tourism studies and in statistical terms (OECD, 2016; Eurostat 2016).

2 In the literature, these activity spaces are often referred to as anchor points (Golledge & Stimson, 1997), bases (Kutter, 1973; Mitchell & Rapkin, 1954), or core stops (Schank & Abelson, 1977).

3 In this paper, the term “mobility” is also used in the plural (“mobilities”), indicating the increasing frequency of locations visited, but also in an attempt to refer to the intertwined nature of physical movements in space with other forms of interactions mediated by ICT.

4 Kaufmann et al. (2004) suggest combining the geographical concept of spatial mobility and the sociological term of social mobility. The focus of sociological applications is foremost on intergenerational (inheritance of the social position of parents by their children) and intragenerational mobility (changes in the social position of individuals over time).

5 Individuals younger than 20 were under‐represented in the database because among the younger age groups, it was the parents who mostly made the contracts with the mobile operator companies. In addition, previous studies have shown that apart from calling and sending messages via mobile phones, other means such as social media channels are used by younger generations in Estonia (see Vihalemm & Kõuts‐Klemm, 2017); data collection methods other than mobile positioning could thus be more appropriate for studying the activity space of respondents under 20.

6 The age distribution of mobile phone users in Telia is similar to those of other operators in the Estonian market (the difference is <2%, except in the age group 20 to 29 years old, whose proportion in the Telia network is 6% higher than other operators).

7 The additional interaction analysis indicates that the higher mobility of the older generation could not be explained by the cross‐border mobility of older Russian speakers. However, previous studies indicate that the character of the contacts varies across ethnic groups, with Russian speakers having more frequent contacts with friends and acquaintances in former Soviet countries (Masso & Tammpuu, 2017).
